# Supplementary material for: Host plant use drives genetic differentiation in syntopic populations of Maculinea alcon
Source: PeerJ. 2016 Mar 31;4:e1865. doi: 10.7717/peerj.1865 (PMC4824886; doi:10.7717/peerj.1865)
Supplement: Supplemental Information 1 — Table S1. Multilocus genotypes of samples at the seven microsatellite loci used for analysis (raw data). Table S2. Summary diversity indices and F-statistics for loci analyzed in Maculinea alcon. Table S3. Linkage (genotypic) disequilibrium for Transylvanian Maculinea alcon populations. Additional Analysis S1. Bayesian clustering of samples using the population assignment programs Structure, BAPS and InStruct. Figure S1. Comparison of the posterior probabilities of the number of clusters (K) identified by the Bayesian population assignment programs Structure, BAPS and InStruct. Figure S2. Bayesian clustering of samples for selected values of K. Figure S3. Matrix plot of principal components. Figure S4. Kinship and half-sibship analysis of samples. Additional Analysis S2. Distribution of Relatedness and Kinship coefficients. Figure S5. Distribution of Relatedness and Kinship coefficients Table S4. Genetic diversity, inbreeding and differentiation when only one individual is selected from each nest. References [file peerj-04-1865-s001.docx]

**Supporting material**

András Tartally, Andreas Kelager, Matthias A. Fürst, David R. Nash

**Host plant use drives genetic differentiation in syntopic populations of *Maculinea alcon***

**Table S1:** Multilocus genotypes of samples at the seven microsatellite loci used for analysis.

**Table S2**: Summary diversity indices and F-statistics for loci analyzed in *Maculinea alcon.*

**Table S3**: Linkage (genotypic) disequilibrium for Transylvanian *Maculinea alcon* populations.

**Additional Analysis S1:** Bayesian clustering of samples using the population assignment programs Structure, BAPS and InStruct.

**Figure S1:** Comparison of the posterior probabilities of the number of clusters (K) identified by the Bayesian population assignment programs Structure, BAPS and InStruct.

**Figure S2:** Bayesian clustering of samples for selected values of K.

**Figure S3:** Matrix plot of principal components.

**Figure S4:** Kinship and half-sibship analysis of samples.

**Additional Analysis S2:** Distribution of Relatedness and Kinship coefficients.

**Figure S5:** Distribution of Relatedness and Kinship coefficients

**Table S4:** Genetic diversity, inbreeding and differentiation when only one individual is selected from each nest.

**References**

**Table S1:** Multilocus genotypes of samples at the seven microsatellite loci used for analysis.

| **Sample ID** | **Site** | ***Myrmica sp.*** | **Nest** | **Year** | **Allele size (bp) - = missing data (locus failed to amplify)** | | | | | | | | | | | | |  |
| --- | --- | --- | --- | --- | --- | --- | --- | --- | --- | --- | --- | --- | --- | --- | --- | --- | --- | --- |
|  |  |  |  |  | **Macu20** | | **Macu26** | | **Macu28** | | **Macu30** | | **Macu31** | | **Macu44** | | **Macu45** | |
| DB15 | Răscruci dry | *scabrinodis* | D sca B | 2009 | 100 | 102 | 108 | 108 | 203 | 209 | 101 | 105 | 129 | 138 | 190 | 208 | 159 | 187 |
| DA14 | Răscruci dry | *sabuleti* | D sab A | 2009 | 96 | 96 | 108 | 114 | 203 | 203 | 101 | 105 | 129 | 129 | - | - | 173 | 185 |
| DD17 | Răscruci dry | *sabuleti* | D sab D | 2009 | 102 | 106 | 108 | 114 | 203 | 203 | 103 | 109 | 129 | 141 | 188 | 202 | 187 | 187 |
| DD18 | Răscruci dry | *sabuleti* | D sab D | 2009 | 100 | 100 | 108 | 108 | 203 | 207 | 101 | 101 | 129 | 129 | 190 | 194 | 171 | 195 |
| DD19 | Răscruci dry | *sabuleti* | D sab D | 2009 | 100 | 102 | 114 | 114 | 203 | 207 | 101 | 103 | 129 | 132 | 188 | 188 | 171 | 195 |
| DD21 | Răscruci dry | *sabuleti* | D sab D | 2009 | 96 | 98 | 114 | 114 | 207 | 207 | 101 | 105 | 129 | 132 | 188 | 190 | 159 | 169 |
| DD22 | Răscruci dry | *sabuleti* | D sab D | 2009 | - | - | 106 | 114 | 203 | 207 | 101 | 103 | 129 | 141 | 190 | 208 | 171 | 203 |
| DD23 | Răscruci dry | *sabuleti* | D sab D | 2009 | 100 | 102 | 108 | 118 | 201 | 215 | 101 | 109 | 129 | 129 | 190 | 194 | 171 | 173 |
| DD24 | Răscruci dry | *sabuleti* | D sab D | 2009 | - | - | 108 | 108 | 205 | 207 | 105 | 109 | 129 | 129 | 194 | 202 | 159 | 177 |
| SAB67-1 | Răscruci dry | *sabuleti* | D SAB67 | 2007 | 100 | 104 | 110 | 114 | 203 | 203 | 101 | 105 | 126 | 129 | 188 | 194 | 173 | 185 |
| SAB67-2 | Răscruci dry | *sabuleti* | D SAB67 | 2007 | 96 | 100 | 108 | 108 | 207 | 211 | 101 | 105 | 129 | 132 | 190 | 194 | 159 | 169 |
| SAB67-3 | Răscruci dry | *sabuleti* | D SAB67 | 2007 | 100 | 104 | 108 | 114 | 199 | 207 | 105 | 109 | 129 | 129 | 188 | 194 | 159 | 185 |
| SAB67-4 | Răscruci dry | *sabuleti* | D SAB67 | 2007 | 100 | 104 | 108 | 114 | 197 | 207 | - | - | 129 | 129 | 188 | 194 | 159 | 171 |
| SAB68 | Răscruci dry | *sabuleti* | D SAB68 | 2007 | 98 | 100 | 114 | 116 | 205 | 211 | - | - | 129 | 129 | 188 | 188 | 179 | 183 |
| SCH66-1 | Răscruci dry | *schencki* | D SCH66 | 2007 | 96 | 100 | 108 | 116 | 207 | 211 | 101 | 109 | 129 | 141 | 190 | 194 | 159 | 169 |
| SCH66-2 | Răscruci dry | *schencki* | D SCH66 | 2007 | 100 | 104 | 108 | 114 | 207 | 207 | 101 | 105 | 129 | 129 | 188 | 188 | 169 | 193 |
| SCH66-3 | Răscruci dry | *schencki* | D SCH66 | 2007 | 100 | 126 | 108 | 114 | 203 | 207 | - | - | 132 | 141 | 188 | 202 | 159 | 159 |
| SCH69-1 | Răscruci dry | *schencki* | D SCH69 | 2007 | 104 | 104 | 108 | 114 | 199 | 211 | - | - | 129 | 129 | 188 | 194 | 181 | 185 |
| SCH69-2 | Răscruci dry | *schencki* | D SCH69 | 2007 | 104 | 104 | 108 | 114 | 205 | 207 | 103 | 105 | 129 | 129 | 184 | 190 | 171 | 171 |
| SCA37 | Răscruci wet | *scabrinodis* | W SCA37 | 2007 | 96 | 96 | 108 | 110 | 203 | 203 | 101 | 101 | 129 | 129 | 190 | 190 | 167 | 167 |
| SCA38 | Răscruci wet | *scabrinodis* | W SCA38 | 2007 | 96 | 102 | 108 | 108 | 195 | 203 | 101 | 107 | 126 | 129 | 190 | 206 | 171 | 179 |
| SCA42 | Răscruci wet | *scabrinodis* | W SCA42 | 2007 | 96 | 102 | 108 | 114 | 203 | 203 | 101 | 101 | 126 | 129 | 188 | 206 | 171 | 171 |
| SCA43 | Răscruci wet | *scabrinodis* | W SCA43 | 2007 | 102 | 102 | 108 | 108 | 203 | 219 | 101 | 107 | 126 | 129 | 190 | 206 | 169 | 171 |
| SCA86-1 | Răscruci wet | *scabrinodis* | W SCA86 | 2007 | 100 | 100 | 108 | 114 | 203 | 207 | 105 | 107 | 126 | 126 | 188 | 190 | 169 | 173 |
| SCA86-2 | Răscruci wet | *scabrinodis* | W SCA86 | 2007 | - | - | 108 | 114 | 203 | 203 | 101 | 105 | 126 | 129 | 188 | 190 | 173 | 185 |
| SCA87-1 | Răscruci wet | *scabrinodis* | W SCA87 | 2007 | - | - | 108 | 108 | 207 | 207 | 101 | 105 | 126 | 129 | 186 | 206 | 171 | 173 |
| SCA87-2 | Răscruci wet | *scabrinodis* | W SCA87 | 2007 | 100 | 102 | 108 | 110 | 203 | 207 | 101 | 105 | 126 | 129 | 190 | 194 | 159 | 197 |
| WA25 | Răscruci wet | *scabrinodis* | W sca A | 2009 | 100 | 102 | 108 | 114 | 203 | 219 | 105 | 107 | 126 | 129 | 190 | 206 | 169 | 179 |
| WA26 | Răscruci wet | *scabrinodis* | W sca A | 2009 | 96 | 102 | 108 | 108 | 205 | 223 | 105 | 105 | 126 | 141 | 190 | 190 | 171 | 177 |
| WB27 | Răscruci wet | *scabrinodis* | W sca B | 2009 | 102 | 102 | 114 | 114 | 203 | 205 | 101 | 105 | 129 | 138 | 188 | 188 | 167 | 171 |
| WB28 | Răscruci wet | *scabrinodis* | W sca B | 2009 | 102 | 102 | 108 | 114 | 203 | 205 | 101 | 105 | 126 | 129 | 188 | 206 | 167 | 171 |
| WB29 | Răscruci wet | *scabrinodis* | W sca B | 2009 | 96 | 102 | 108 | 114 | 203 | 211 | 101 | 105 | 129 | 138 | 188 | 190 | 171 | 171 |
| WB30 | Răscruci wet | *scabrinodis* | W sca B | 2009 | 96 | 102 | 108 | 114 | 203 | 223 | 101 | 105 | 129 | 138 | 188 | 206 | 171 | 177 |
| WB31 | Răscruci wet | *scabrinodis* | W sca B | 2009 | 102 | 102 | 108 | 114 | 203 | 205 | 101 | 105 | 126 | 129 | 188 | 206 | - | - |
| WC32 | Răscruci wet | *scabrinodis* | W sca C | 2009 | 96 | 102 | - | - | 195 | 203 | 101 | 101 | 126 | 129 | 206 | 206 | 167 | 179 |
| WC33 | Răscruci wet | *scabrinodis* | W sca C | 2009 | 96 | 100 | 108 | 108 | 195 | 203 | 101 | 101 | 126 | 129 | 190 | 206 | 167 | 179 |
| WC34 | Răscruci wet | *scabrinodis* | W sca C | 2009 | 96 | 100 | 108 | 108 | 195 | 203 | 101 | 105 | 129 | 129 | 190 | 206 | 167 | 179 |
| WD35 | Răscruci wet | *scabrinodis* | W sca D | 2009 | 102 | 102 | 108 | 108 | 203 | 223 | 101 | 105 | 126 | 138 | 190 | 206 | 171 | 177 |
| WD36 | Răscruci wet | *scabrinodis* | W sca D | 2009 | 102 | 102 | 108 | 114 | 203 | 223 | 101 | 105 | 126 | 138 | 188 | 206 | - | - |
| WD37 | Răscruci wet | *scabrinodis* | W sca D | 2009 | 102 | 102 | 108 | 108 | 203 | 223 | - | - | 126 | 126 | 190 | 206 | 171 | 177 |
| WE38 | Răscruci wet | *scabrinodis* | W sca E | 2009 | 96 | 96 | 108 | 110 | 201 | 203 | 101 | 105 | 126 | 129 | 196 | 206 | 167 | 175 |
| WE39 | Răscruci wet | *scabrinodis* | W sca E | 2009 | 96 | 100 | 108 | 110 | 201 | 219 | 101 | 105 | 126 | 129 | 190 | 208 | 171 | 175 |
| WE40 | Răscruci wet | *scabrinodis* | W sca E | 2009 | 96 | 96 | 110 | 114 | 201 | 219 | 101 | 105 | 129 | 129 | 190 | 206 | 169 | 175 |
| WE41 | Răscruci wet | *scabrinodis* | W sca E | 2009 | 96 | 96 | 108 | 108 | 195 | 203 | 101 | 105 | 126 | 129 | 190 | 206 | 167 | 171 |
| WE42 | Răscruci wet | *scabrinodis* | W sca E | 2009 | 92 | 96 | 108 | 110 | 201 | 203 | 105 | 105 | 126 | 129 | 190 | 196 | 167 | 175 |
| WE43 | Răscruci wet | *scabrinodis* | W sca E | 2009 | 100 | 102 | 110 | 114 | 201 | 207 | 101 | 105 | 126 | 126 | 190 | 206 | 171 | 175 |
| WE44 | Răscruci wet | *scabrinodis* | W sca E | 2009 | 96 | 96 | 110 | 110 | 203 | 219 | 105 | 107 | 129 | 129 | 190 | 208 | 169 | 179 |
| SA01 | Şardu | *scabrinodis* | S sca A | 2009 | 100 | 100 | 110 | 114 | 203 | 207 | 105 | 105 | 129 | 132 | 188 | 194 | 163 | 179 |
| SA02 | Şardu | *scabrinodis* | S sca A | 2009 | 100 | 100 | 110 | 114 | 207 | 223 | 105 | 105 | 126 | 138 | 188 | 194 | 163 | 191 |
| SA03 | Şardu | *scabrinodis* | S sca A | 2009 | 100 | 100 | 108 | 110 | 203 | 215 | 101 | 105 | 129 | 129 | 194 | 198 | 159 | 175 |
| SA04 | Şardu | *scabrinodis* | S sca A | 2009 | 100 | 100 | 108 | 108 | 223 | 223 | 103 | 103 | 126 | 129 | 186 | 190 | 171 | 171 |
| SB05 | Şardu | *scabrinodis* | S sca B | 2009 | 100 | 100 | 108 | 114 | 203 | 203 | 101 | 103 | 129 | 132 | 188 | 190 | 167 | 179 |
| SB06 | Şardu | *scabrinodis* | S sca B | 2009 | 100 | 100 | 108 | 128 | 203 | 203 | 101 | 103 | 126 | 129 | 188 | 190 | 167 | 179 |
| SB07 | Şardu | *scabrinodis* | S sca B | 2009 | 100 | 100 | 108 | 114 | 203 | 215 | 101 | 103 | 126 | 138 | 188 | 206 | 169 | 179 |
| SC08 | Şardu | *scabrinodis* | S sca C | 2009 | 98 | 122 | 110 | 114 | 203 | 205 | 105 | 105 | 126 | 138 | 194 | 206 | 159 | 169 |
| SC09 | Şardu | *scabrinodis* | S sca C | 2009 | 98 | 122 | 110 | 114 | 203 | 207 | 101 | 105 | 129 | 138 | 194 | 206 | 159 | 179 |
| SD10 | Şardu | *scabrinodis* | S sca D | 2009 | 100 | 102 | 108 | 114 | 203 | 207 | 105 | 105 | 129 | 132 | 188 | 206 | 155 | 181 |
| SF13 | Şardu | *scabrinodis* | S sca F | 2009 | 100 | 100 | 106 | 108 | 203 | 207 | 105 | 105 | 129 | 132 | 186 | 188 | 207 | 207 |
| SE11 | Şardu | *vandeli* | S van E | 2009 | 100 | 100 | 108 | 110 | 195 | 215 | 101 | 105 | 126 | 129 | 190 | 194 | 159 | 179 |
| SE12 | Şardu | *vandeli* | S van E | 2009 | 100 | 100 | 106 | 114 | 195 | 215 | 101 | 105 | 126 | 132 | 188 | 198 | 155 | 181 |

**Table S2**: **Summary diversity indices and *F*-statistics for loci analyzed in *Maculinea alcon***. Locus name; N_avg_: average number of individuals per locus; *A*, number of alleles; *A*_R_, allelic richness adjusted for sample size using rarefaction (based on a minimum sample size of 13 individuals); *H*_O_, average observed heterozygosity; *H*_S_, Nei’s unbiased expected within-population heterozygosity; *H*_T_, Nei’s overall heterozygosity; *f* (*F*_IS_), Weir & Cockerham’s inbreeding coefficient; *θ* (*F*_ST_), Weir & Cockerham’s genetic differentiation; *G*_ST_': Nei's sample independent genetic differentiation, *G*''_ST_: Meirman & Hedrick's sample-independent standardized genetic differentiation, *D*_EST_: Jost's genetic differentiation. Values significantly different from zero (based on Bonferroni correction for individual loci) are shown in bold.

| Locus | *N_avg_* | *A* | *A*_R_ | *H*_O_ | *H*_S_ | *H*_T_ | *f* (*F*_IS_) | *θ* (*F*_ST_) | *G*_ST_' | *G*''_ST_ | *D*_EST_ |
| --- | --- | --- | --- | --- | --- | --- | --- | --- | --- | --- | --- |
| Macu20 | 18.7 | 9 | 5.65 | 0.498 | 0.595 | 0.726 | 0.166 | 0.241 | **0.248** | **0.781** | **0.588** |
| Macu26 | 19.7 | 7 | 4.35 | 0.733 | 0.650 | 0.663 | -0.103 | 0.030 | 0.029 | 0.193 | 0.120 |
| Macu28 | 20.0 | 12 | 8.30 | 0.788 | 0.766 | 0.786 | -0.048 | 0.044 | 0.038 | **0.414** | **0.313** |
| Macu29^1^ | 17.0 | 15 | 8.74^α^ | 0.513 | 0.845 | 0.885 | **0.379** | - | - | - | - |
| Macu30 | 18.3 | 5 | 4.42 | 0.750 | 0.644 | 0.665 | -0.209 | 0.048 | 0.046 | **0.391** | **0.253** |
| Macu31 | 20.0 | 5 | 4.53 | 0.716 | 0.595 | 0.647 | -0.205 | 0.125 | **0.117** | 0.206 | 0.130 |
| Macu44 | 19.7 | 10 | 6.69 | 0.897 | 0.768 | 0.805 | -0.167 | 0.083 | **0.069** | **0.424** | **0.336** |
| Macu45 | 19.3 | 20 | 11.68 | 0.858 | 0.883 | 0.910 | 0.014 | 0.047 | **0.044** | **0.713** | **0.652** |
| Overall^β^ | 19.1 | 9.71 | 6.66 | 0.744 | 0.697 | 0.751 | -0.074 | **0.090** | **0.085** | **0.461** | **0.336** |

^α^ *A*_R_ based on minimum sample size of 11 individuals

^β^ Locus Macu29 excluded

^1^ Excluded due to suspected null-alleles

**Table S3**: **Linkage (genotypic) disequilibrium for Transylvanian *Maculinea alcon* populations**. *P*-values are based on 1,680 permutations. The Bonferroni adjusted *P*-value for 5 % nominal level was 0.0006. Bold values were significant after sequential Bonferroni correction.

| **Locus pairs** | **Răs_dry** | **Răs_wet** | **Şardu** | **Overall** |
| --- | --- | --- | --- | --- |
| Macu20 × Macu26 | 0.2393 | 0.2429 | 0.8262 | 0.2589 |
| Macu20 × Macu28 | 0.5887 | 0.0387 | 0.7512 | 0.0845 |
| Macu20 × Macu29 | 0.0905 | 0.0024 | 0.5161 | **0.0006** |
| Macu20 × Macu30 | 0.7571 | 0.5435 | 1.0000 | 0.7327 |
| Macu20 × Macu31 | 0.3970 | 0.4268 | 0.6679 | 0.3417 |
| Macu20 × Macu44 | 0.0292 | 0.1441 | 0.4042 | 0.0125 |
| Macu20 × Macu45 | 0.2810 | 0.0208 | 0.9780 | 0.0500 |
| Macu26 × Macu28 | 0.9637 | 0.0310 | 0.7655 | 0.1821 |
| Macu26 × Macu29 | 0.3327 | 0.3720 | 1.0000 | 0.2607 |
| Macu26 × Macu30 | 0.8911 | 0.5012 | 0.0524 | 0.3316 |
| Macu26 × Macu31 | 0.0762 | 0.3696 | 0.4458 | 0.0810 |
| Macu26 × Macu44 | 0.2167 | **0.0006** | 0.0024 | **0.0006** |
| Macu26 × Macu45 | 1.0000 | 0.1036 | 1.0000 | 0.3554 |
| Macu28 × Macu29 | 0.5607 | 0.0077 | 1.0000 | 0.0137 |
| Macu28 × Macu30 | 0.0542 | 0.1750 | 0.0280 | 0.0060 |
| Macu28 × Macu31 | 0.9970 | 0.1643 | 0.1298 | 0.4036 |
| Macu28 × Macu44 | 0.7417 | 0.0066 | 0.4030 | 0.0155 |
| Macu28 × Macu45 | 0.0012 | **0.0006** | 0.2970 | **0.0006** |
| Macu29 × Macu30 | 0.6155 | 0.7929 | 1.0000 | 0.7643 |
| Macu29 × Macu31 | 0.5792 | 0.7268 | 1.0000 | 0.7089 |
| Macu29 × Macu44 | 0.1732 | 0.6827 | 1.0000 | 0.3917 |
| Macu29 × Macu45 | 0.2702 | 0.0113 | 1.0000 | 0.0060 |
| Macu30 × Macu31 | 0.8696 | 0.4869 | 0.2958 | 0.6077 |
| Macu30 × Macu44 | 0.8768 | 0.6839 | 0.2524 | 0.5649 |
| Macu30 × Macu45 | 0.0667 | 0.1982 | 0.1369 | 0.0137 |
| Macu31 × Macu44 | 0.5893 | 0.8298 | 1.0000 | 0.8458 |
| Macu31 × Macu45 | 0.8446 | 0.9316 | 1.0000 | 0.9500 |
| Macu44 × Macu45 | 0.4119 | 0.7583 | 0.1399 | 0.3286 |

**Additional Analysis S1: Bayesian clustering of samples using the population assignment programs Structure, BAPS and InStruct**.

In addition to analyzing the population structure of the samples using Structure (see main text), we repeated the analysis using the software packages BAPS version 5.2 (Corander et al. 2008b), and InStruct version 1.0 (Gao et al. 2007), the last of which does not assume Hardy-Weinberg equilibrium within clusters

In baps, the upper bound to number of samples was set to 60 (*N*_individuals_ = 60) with 10 repetitions, when employing the generic learning clustering method setting. Log(ml) values for individual mixture clustering were averaged over the 10 best visited partitions over 100 runs using the fixed *K* mode. The level of admixture was examined based on the individual clustering mixture analysis for the most likely *K*, allowing 1 individual to define a population but otherwise using the parameter settings recommended by Corander et al. (2008a): 100 iterations, 200 reference samples and 10 iterations for reference individuals. In Structure,

In InStruct, 10 chains were used, with a burn-in length of 50,000 MCMCs and a simulation run of 1,000,000 MCMCs, and the option was used to infer both population structure and the inbreeding coefficients for subpopulations.

For BAPS, the most likely value of K was based on the maximum log-likelihood. For InStruct, the most likely value of *K* was chosen based on the deviance information criterion, but the ∆*K* method of Evanno et al. (2005) was also applied for comparison.

*Results*

BAPS analysis showed similar log-likelihood values for *K* = 8-11, where *K* = 9 and *K* = 10 were found to be equally probable (Figure S1). The cluster membership of samples were explored for K = 2-5 and K = 9-11 and, for the lower Ks, revealed a high degree of similarity with the result from the Structure analysis (Figures 1, S2). For the higher *K*s, allowing a much greater substructuring, nests within designated populations seemed to explain much of the cluster membership, particularly so for Răscruci wet site, which also appeared more genetically homogenous as they were generally assigned to the same two clusters (Figure S2).

InStruct revealed the same general pattern as Structure and BAPS (Figures 1, S1, S2), with the most likely estimate based on the deviance information criterion being K = 10 (Figure S2). Estimated within-subpopulation inbreeding coefficients were generally low at low K (F_IS_ = 0.025 for K = 2), but as expected, were higher at higher values of K (F_IS_ = 0.033-0.495 for K = 10), reflecting greater population sub-structure.

**Figure S1: Comparison of the posterior probabilities of the number of clusters (K) identified by the Bayesian population assignment programs Structure, BAPS and InStruct**. Each line shows the mean posterior probability for each value of K across simulations, with error bars representing the standard deviation across simulations. Also shown are the ΔK values of the posterior probabilities from Structure and InStruct using the method of (Evanno et al. 2005) shown relative to the maximum value of ΔK. Peaks in the value of ΔK may represent different levels of population substructure.

**
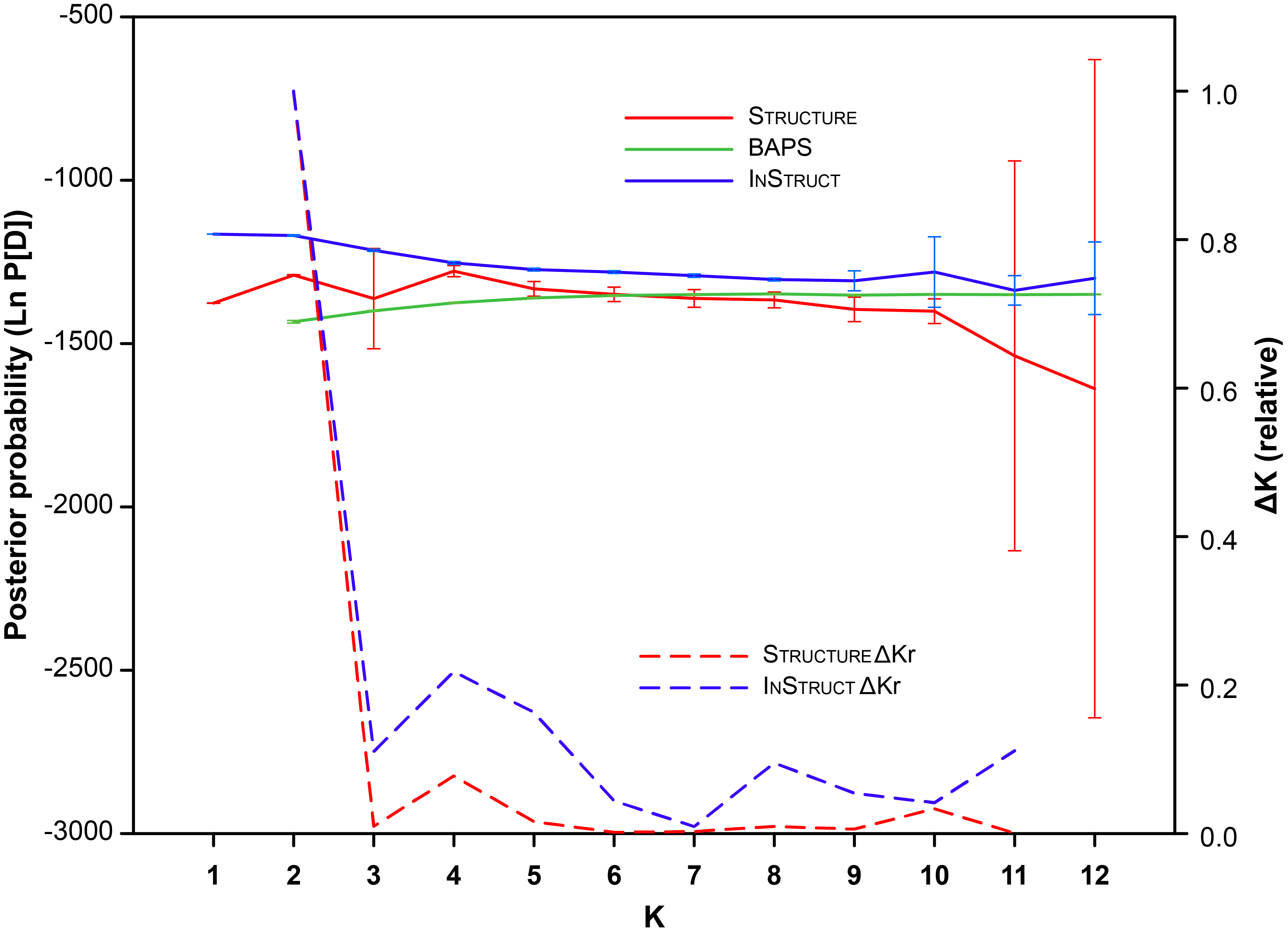
**

**Figure S2: Bayesian clustering of samples for selected values of K.** Comparison of genetic clustering of samples into groups using the Bayesian clustering programs Structure, BAPS and InStruct based on the most likely number of clusters identified by each program (Fig. S1 and main text). Each column represents an individual, and is divided according to its probability of membership of different clusters, which are represented by different colours.


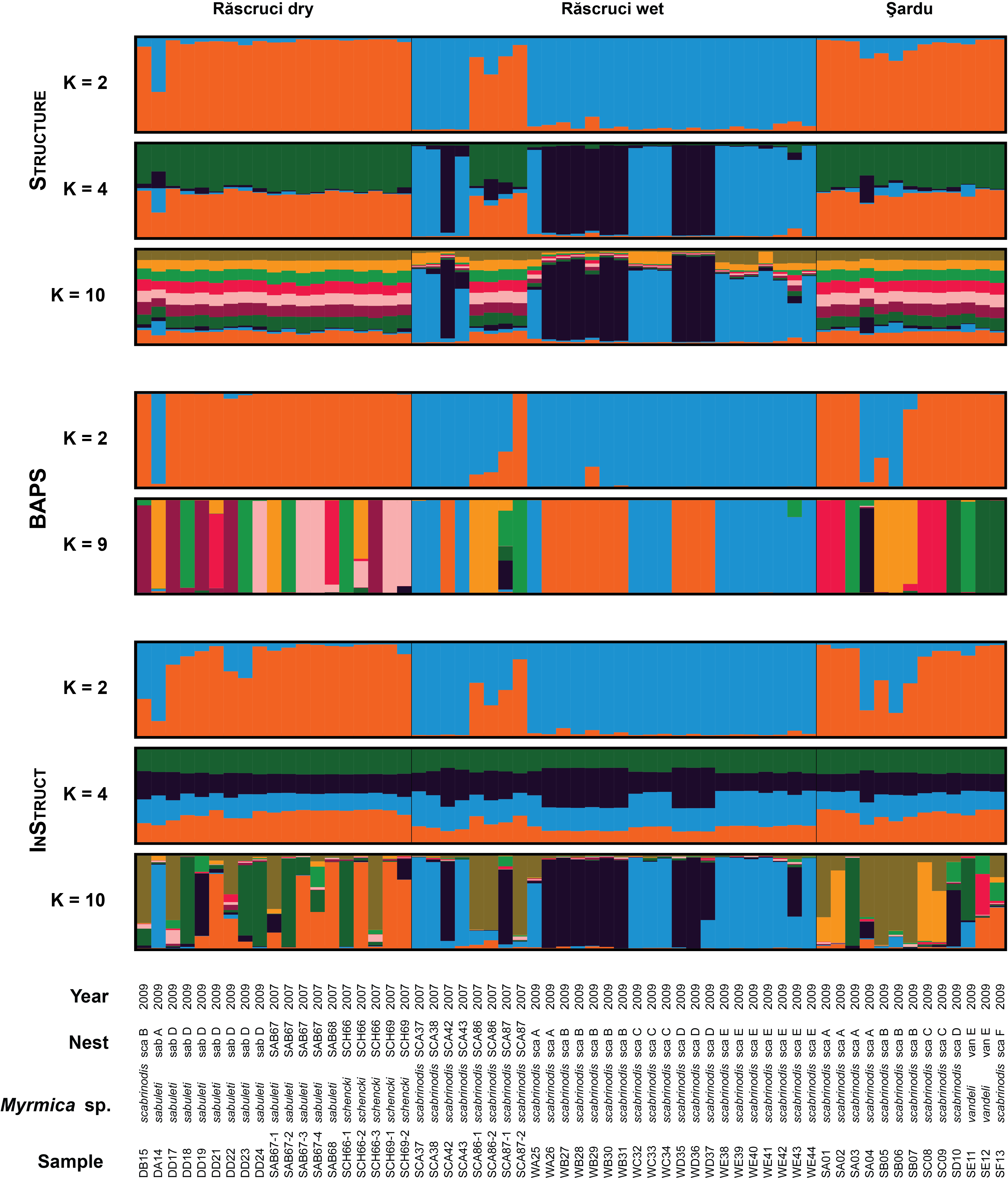


**Figure S3: Matrix plot of principal components.** Each panel shows an ordination plot pairs of principal components. Each symbol represents an individual, coloured according to its pre-defined population (blue = Răscruci wet, orange = Răscruci dry, purple = Şardu). Coloured regions are convex hulls enclosing all samples from each pre-defined population. The proportion of the total variance explained by each principal component is shown in parentheses. The upper left plot is shown in more detail in Figure 2 of the main text.

**
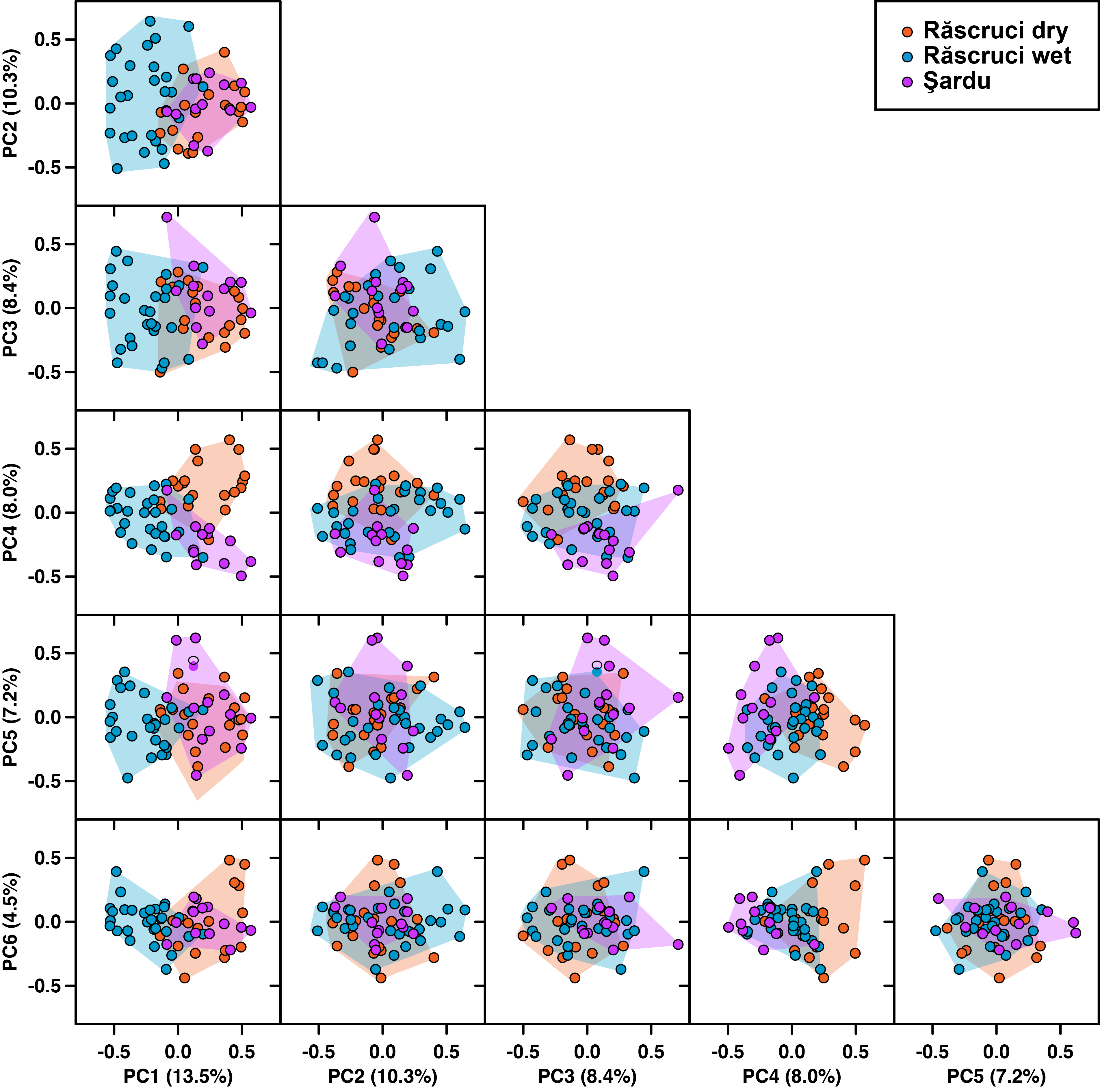
**

**Figure S4: Kinship and half-sibship analysis of samples.** The pairwise matrix shows the estimated kinship coefficient (Queller & Goodnight 1989) of each pair of individuals (excluding those with negative kinship; see figure S4) above the diagonal, and the probability that each pair are half siblings based on maximum likelihood estimates from Colony (Jones & Wang 2010) below the diagonal. Comparisons between samples from the same pre-defined population are shaded according to the same colour scheme as Figure 2 (blue = Răscruci wet, orange = Răscruci dry, purple = Şardu). Individuals sharing the same ant nest are outlined with lines in these same colours, and those sharing the same *Myrmica* species as host are outlines with black lines. The area and shade of each data point is proportional to the relatedness or probability of being full siblings for that pair of individuals.


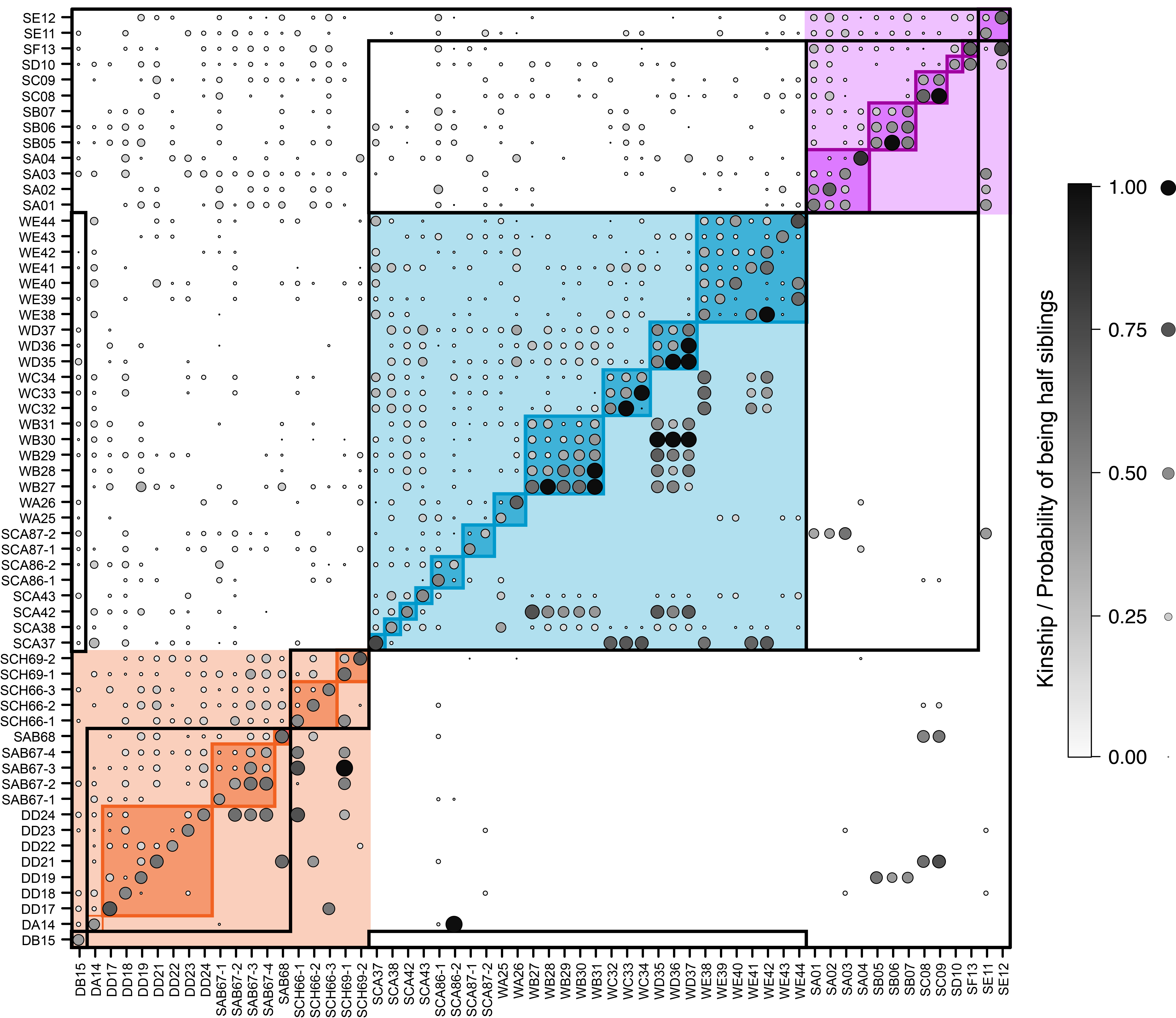


**Additional Analysis S2: Distribution of Relatedness and Kinship coefficients**

The distribution of pairwise relatedness (Queller & Goodnight 1989) and kinship (Loiselle et al. 1995) values between individuals was further examined by dividing pairs into different groups depending on whether they shared the same nest, the same host ant species and the same site (Figure S5). In addition, kinship coefficients comparing each individual with itself were included in a separate group.

Whether groups differed in their relatedness of kinship coefficients was explored using pairwise PERMANOVA (permutational ANOVA) between groups as implemented in Past 3.11 (Hammer et al. 2001), with 10,000 unrestricted permutations, which yields a *Pseudo-F* value comparable to ANOVA (c.f. Iacchei et al. 2013). Bonferroni correction was applied to the obtained *P*-values to account for multiple comparisons.

***Results***

There was no significant difference between either relatedness or kinship coefficients when individuals from different sites were compared, regardless of which host ant species they were collected from (*Pseudo-F* = 0.854, *P* > 0.999; *Pseudo-F* = 1.038, *P* > 0.999 respectively). Individuals collected from the same site had significantly higher relatedness and kinship that those collected from different sites (All *Pseudo-F* > 50*,* *P* < 0.002). For relatedness, there was no significant difference between individuals collected from different nests within each site, regardless of which host ant species they exploited (*Pseudo-F* = 0.037, *P* = 0.844), but individuals collected from different nests of the same host ant species showed *lower* pairwise kinship than those collected from the nests of different species, a comparison that was just significant (*Pseudo-F* = 10.28, *P* = 0.032). Individuals collected from the same nest had significantly higher pairwise relatedness than those collected from other nests (All *Pseudo-F* > 14*,* *P* < 0.003), and the median values for these comparisons fell within those expected for half-siblings (Figure S5). The highest values of kinship (All *Pseudo-F* > 256*,* *P* < 0.0015) were found, unsurprisingly, when individuals were compared with themselves.

**Figure S5: Distribution of A) Relatedness and B) Kinship coefficients.** Histograms represent the distribution of coefficients inn pairwise comparisons between individuals, divided according to the origin of the compared individuals. Note that the frequencies of the different comparisons are very different, as reflected in the frequency axis on the right hand side of the figure. Background shading reflects the expected value for different types of relationship. Lower-case letters to the right of each histogram area shared by distributions that are not significantly different based on PERMANOVA.

**Table S4: Genetic diversity, inbreeding and differentiation when only one individual is selected from each nest.** The table below repeats the analyses presented in table 3 of the main text, but selected only a single sample from each *Myrmica* nest. This selection was carried out randomly using the “special include” option of GenoDive (Meirmans & Van Tienderen 2004) to produce ten datasets with different samples included. Values above the diagonal in the matrix (with blue background) are mean values of *θ* (*F*_ST_) ± SE across these ten datasets, values along the diagonal (with green background) are *F*_IS_ ± SE, and values below the diagonal (with yellow background) are *G''*_ST_ ± SE / *D*_EST_ ± SE. Values in bold differ significantly from zero (median *P* < 0.05). Below the matrix are mean values of four different measures of within-population genetic diversity. The effective number of alleles per locus (*A*_E_), the observed heterozygosity (*H*_O_), the expected heterozygosity (*H*_E_) and the unbiased expected heterozygosity (*uH*_E_). These values are based on mean values per locus across the ten datasets, with SE across datasets in parentheses and ± SE across loci. *P*-values for comparisons between pre-defined populations based on mixed model comparison across loci are shown on the right.

Values are generally similar to those obtained for the whole dataset, which are contained within the standard errors of the mean, except for the effective number of alleles, where the reduced sample size has led to a consistently lower effective number of alleles.

|  | Răscruci dry | Răscruci wet | Şardu |  |
| --- | --- | --- | --- | --- |
| Răscruci dry | -0.014 ± 0.015 | **0.078 ± 0.004** | **0.046 ± 0.005** |  |
| Răscruci wet | **0.185 ± 0.014 / 0.247 ± 0.017** | -0.079 ± 0.018 | **0.116 ± 0.005** |  |
| Şardu | **0.126 ± 0.018 /**  **0.165 ± 0.023** | **0.276 ± 0.016 /**  **0.358 ± 0.018** | -0.088 ± 0.021 |  |
|  |  |  |  | *P* |
| *A*_E_ | 3.783 (0.072) ± 0.609 | 2.925 (0.037) ± 0.424 | 3.518 (0.071) ± 0.616 | 0.178 |
| *H*_O_ | 0.744 (0.012) ± 0.071 | 0.704 (0.009) ± 0.040 | 0.770 (0.010) ± 0.103 | 0.828 |
| *H*_E_ | 0.678 (0.005) ± 0.061 | 0.623 (0.004) ± 0.039 | 0.656 (0.004) ± 0.058 | 0.687 |
| *uH*_E_ | 0.737 (0.006) ± 0.066 | 0.655 (0.005) ± 0.041 | 0.714 (0.004) ± 0.064 | 0.494 |

**References**

Corander J, Marttinen P, Sirén J, and Tang J. 2008a. *BAPS: Bayesian Analysis of Population Structure - Manual (version 5.2).*

Corander J, Marttinen P, Sirén J, and Tang J. 2008b. Enhanced Bayesian modelling in BAPS software for learning genetic structures of populations. *BMC Bioinformatics* 9:539. 10.1186/1471-2105-9-539

Evanno G, Regnaut S, and Goudet J. 2005. Detecting the number of clusters of individuals using the software STRUCTURE: a simulation study. *Molecular Ecology* 14:2611-2620.

Gao H, Williamson S, and Bustamante CD. 2007. A Markov chain Monte Carlo approach for joint inference of population structure and inbreeding rates from multilocus genotype data. *Genetics* 176:1635-1651. 10.1534/genetics.107.072371

Hammer Ø, Harper DAT, and Ryan PD. 2001. PAST: Paleontological Statistics Software Package for education and data analysis. *Palaeontologia Electronica* 4:1-9.

Iacchei M, Ben-Horin T, Selkoe KA, Bird CE, García-Rodríguez FJ, and Toonen RJ. 2013. Combined analyses of kinship and FST suggest potential drivers of chaotic genetic patchiness in high gene-flow populations. *Molecular Ecology* 22:3476-3494. 10.1111/mec.12341

Jones OR, and Wang J. 2010. COLONY: a program for parentage and sibship inference from multilocus genotype data. *Molecular Ecology Resources* 10:551-555. 10.1111/j.1755-0998.2009.02787.x

Loiselle BA, Sork VL, Nason J, and Graham C. 1995. Spatial genetic structure of a tropical understory shrub, Psychotria officinalis (Rubiaceae). *American Journal of Botany* 82:1420-1425.

Meirmans PG, and Van Tienderen PH. 2004. GENOTYPE and GENODIVE: two programs for the analysis of genetic diversity of asexual organisms. *Molecular Ecology Notes* 4:792-794. 10.1111/j.1471-8286.2004.00770.x

Queller D, and Goodnight K. 1989. Estimating relatedness using genetic markers. *Evolution* 43:258-275.
